# Power of counter movement jumps with external load – coherence of three assessment methods

**DOI:** 10.1186/s13104-015-1122-z

**Published:** 2015-04-16

**Authors:** Marie Hilmersson, Ida Edvardsson, Åsa B Tornberg

**Affiliations:** Surgical and Preoperative Science, Sports Medicine, Umeå University, Umeå, Sweden; Department of Health Sciences, Physiotherapy, Lund University, Baravägen 3, 221 85 Lund, Sweden; Genetic & Molecular Epidemiology (GAME) Unit, Lund University Diabetes Center (LUDC) Clinical Research Center, Skåne University Hospital, Malmö, Sweden

**Keywords:** Muscle strength, Power, Neuromuscular capacity, Jump ability, Reliability

## Abstract

**Background:**

The purpose of this study was to evaluate the coherence between three different methods assessing the power driven from a counter movement jump (CMJ); the Powertimer 300-series contact mat (C-mat), the MuscleLab 4010 infrared mat (IR-mat) and the MuscleLab 4010 linear encoder (M-encoder), and to evaluate the test-retest reliability of the M-encoder.

**Methods:**

Twenty-two males and 29 female, elite athletes performed two test sessions with three days in between. Each test session included counter movement jumps (CMJ) performed on a Smith-machine with external loads of 40 kg. Jump height and flight time were assessed with C-mat and IR-mat, and power was additionally assessed with C-mat. Variables analyzed from the M-encoder were average power (AP), average force (AV), average velocity (AV), and distance (D).

**Results:**

The results from the C-mat were systematically higher than the ones obtained from the M-encoder and IR-mat. The correlation between the C-mat, M-encoder and the IR-mat was strong (r_p_ = 0.95-0.98). The results showed a high test-retest reliability for all indices assessed with the M-encoder, AP (r_p_ = 0.97, p < 0.001; TE% = 3.9%), AF (r_p_ = 0.99, p < 0.001; TE% = 1.4%). Furthermore, the AV had high values (r_p_ = 0.94, p < 0.001; TE% = 2.9%) as well as D (r_p_ = 0.87, p < 0.001; TE% = 5.4%).

**Conclusion:**

It is important to use the same equipment in both pre- and post-testing, since all three methods were reliable, coherent but not interchangeable to each other.

## Background

In sport science, aiming at enhancing elite athlete performance through exercise-training, accurate methods for testing the performance are important [1]. Performance tests need to be valid, reliable, and sensitive to be able to detect the smallest meaningful changes due to exercise-training [1]. Elite athletes need to test their progress of exercise-training on a regular basis and it is therefore very important that the test methodology is reproducible, and that it is associated with a small within-subject variation [2]. Measurement error occurs during all types of testing and thereby will the test-retest reliability be very important to analyze, since it demonstrates the reproducibility of repeated measurements.

Generating high power is important for many elite athletes and the use of loaded vertical jumps as an exercise-training method has been shown to be effective to increase muscular strength and power [3,4]. Vertical jumps are also commonly used to assess an individual’s muscular strength and power [3,5,6]. Countermovement jump (CMJ) is one of the most commonly used vertical jump techniques to evaluate muscle strength, power, and jump height in athletes [3,7,8], and have been shown to be reliable during assessments of vertical jump power [9,10].

Equipment widely used for testing muscular strength, power, and jump height are different types of contact mats [8,10,11], infrared mats [12], force platforms [3,7,9], and position transducer [7]. It is important to analyze the validity and reliability of all equipment used during these assessments, before using them to test muscular strength, power, and jump height. The knowledge of coherence between different assessment systems is important if they are to be used interchangeably. The contact mat (C-mat) (Powertimer300-serie, Newtest, Oulu, Finland) has been validated previously [10], but the coherence with MuscleLab 4010 linear encoder (M-encoder) (Ergotest Innovation, Langensund, Norway) and MuscleLab 4010 infrared mat (IR-mat) (Ergotest Innovation, Langensund, Norway) has, to the best of our knowledge, not been tested. The validity of the C-mat was analyzed by comparing the assessments of jump height with the assessments of jump height from a force platform [10]. These assessments showed that the C-mat assessed higher jump heights compared to the force platform, with a systematic bias for CMJ (2.8 cm) and squat jump (1.7 cm). The C-mat was also shown to be reliable [10]. The IR-mat has been compared with other infrared mats and the analysis showed that the two optical timing systems can be used interchangeably [12]. The M-encoder is a new way of assessing power during sport performance by means of measuring the velocity of weight displacement and need thereby to be tested regarding reliability and coherence.

Several studies involving coherence, reliability, and CMJ have been conducted on contact mats and force platforms [3,7,9]. However, to the best of our knowledge, there are no studies evaluating the coherence of C-mat, IR-mat, and M-encoder, which we therefore sought to examine. A second aim was to evaluate the test-retest reliability of the M-encoder assessing loaded CMJ.

## Methods

### Subjects

Fifty-one individuals were recruited into this study. All of the subjects were team members of different teams at the highest leagues in Sweden, and thereby considered to be elite athletes. The participants consisted of athletes from football, basketball, volleyball, ice hockey, handball, and track and field sports. All participants were given an oral and written description of the test and signed an informed consent. Ethical principles outlined in the declaration of Helsinki were followed. This study was approved by the Ethics Committee in Lund, Sweden (ETIK 2009/699).

Participants in the coherence analysis (session 1) were 22 men and 29 women. The men’s age, mass, and height were 22.5(4.7) years, 83.8(13.9) kg, and 184(8) cm, respectively. The women’s age, mass, and height were 20.3(3.2) years, 68(7.4) kg, and 174(6) cm, respectively. The external loading of 40 kg was 50(8) % and 59(6) % respectively.

Participants in the test-retest analysis (session 1 and session 2) were 18 men and 23 women completed the tests on both test session one and two. The men’s age, mass, and height were 21.8(4.3) years, 81.9(13.7) kg, and 183(8) cm, respectively. The women’s age, mass, and height were 20.3(3.2) years, 68(7.4) kg, and 173(6) cm, respectively. The external loading of 40 kg was 50(8) % and 59(6) % respectively.

### Procedure

Test-retest CMJ with external load was performed on a Smith machine (Nordic Gym, Bollnäs, Sweden) with a three-day interval between tests to evaluate the test-retest reliability. The participants were informed to refrain from eating, drinking coffee, or smoking two hours before each test session. They were also informed to refrain from performing any heavy exercise 48 hours prior to the tests. Before testing, each person answered questions concerning their health and training status. Then they were weighted on an electronic glass scale (OBH Nordica, light line 6251, Spånga, Sweden) before they performed a ten-minute sub-maximal warm-up on a bicycle with a workload of 1 W∙kg^−1^ body weight (Monark, ergomedic 828E, Varberg, Sweden). This was followed by a familiarization session of three jump trails at a sub-maximal level. During testing, verbal encouragement was used for all persons for each attempt. Two investigators administered all tests, but were responsible for different tasks and equipment in a standardized fashion.

After the three jump trails of familiarization, the test procedure started. The different loads for the men were 20, 40, 60, 80 and 100 kg and for the women 20, 30, 40, 50 and 60 kg. Each subject performed three jump trails on both legs with a barbell on their shoulders connected to a Smith machine. A three-minute rest followed the three jump trails on each load. The load was chosen because it is a common load used in our lab during testing of elite athletes. 40 kg was chosen to analyze the reliability and coherence at the same absolute external load for both men and women. Before the actual test started, the participants were told to bend their knees to about 90 degrees, which was marked on the Smith machine and measured with a conventional goniometer. At the same time, marks were placed for the hand and foot positions. During the CMJ, the participants completed a fast downward movement followed by a fast upward movement when the barbell reached the marking on the smith machine. The participants were given verbal guidance concerning the positions. The three trails during test session 1 were used in the intra-session analysis and the best jump was used for the evaluation of the inter-session and the coherence analyzes. The equipment used for measurement during all jumps was the M-encoder, IR-mat, and C-mat. M-encoder measured the average power (AP). IR-mat measured jump height and flight time. C-mat measured power, jump height, and flight time. The M-encoder was used for measures of average power (AP), average force (AF), average velocity (AV), and distance (D). To be able to analyze the knee angle in Dartfish (version 4.5.1.0, Fribourg, Switzerland), the jumps were recorded from the left side with a digital video camera (Panasonic NV-GS230, Osaka, Japan). To make the analyses more convenient, tape markings were placed on trochanter major, the lateral condyle and just above the lateral malleolus of the fibula on the left leg.

### Instruments

*C-mat* (Powertimer 300-series, Newtest, Tyrnävä, Finland) a contact mat assessing flight time from when the subject’s foot leaves the contact mat until the foot touches the mat again. The jump height was calculated as formula jh = (g∙tf)^2^/8 (jh = jump height, g = 9.81 m/s^2^ gravitation, and tf = flight time) and power was calculated as P = 60.7∙jh + 45.3 ∙bm-2055 (P = power, jh = jump height and bm = body mass) using the software handed by the manufacturer.

*IR-mat* (MuscleLab 4010, Ergotest Innovation, Langensund, Norway) an infrared mat assessing flight time from when the subject’s foot leaves the infrared beam until the foot crosses the beam again. Jump height was calculated by the software handed by the manufacturer using the formula jh = (g∙tf)^2^/8 (jh = jump height, g = 9,81 m/s^2^ gravitation, and tf = flight time).

*M-encoder* (MuscleLab 4010, Ergotest Innovation, Langensund, Norway) a linear encoder assessing speed and acceleration of the barbell through a wire attached to the barbell. Average power was calculated by the software handed by the manufacturer using the formula P = F∙v (P = power, F = force, and v = velocity). Average force was calculated by the software handed by the manufacturer using the formula F = m∙g + m∙ a (g = 9.81 m∙s^−2^ gravitation, m = mass kg, and a = acceleration m∙s^−2^).

### Statistics

The Kolmogorov-Smirnov test was used to test the normal distribution of the data. Values throughout are given as means and standards deviations (SD). Comparing the results between session 1 and 2 for the different gendes, a 2 (gender) x 2 (session) analyzes was used with a repeated measures ANOVA approach. The gender (n = 2) and the sessions (n = 2) were considered as the within-participant factor. Greenhouse-Geisser correction was used and the Sidak adjustment was applied during the post hoc analysis for multiple comparisons.

Intra-session reliability 2 (methods) x 3 (trails) was analyzed with a repeated measures ANOVA approach. The methods (n = 2) and the trials (n = 3) were considered as the within-participant factor. Greenhouse-Geisser correction was used and the Sidak adjustment was applied during the post hoc analysis for multiple comparisons. The choice of statistical approach was in agreement with Marina and Torrado [13].

Test-retest correlations were calculated both for intra-session and inter-session relations. Intraclass correlation coefficient (ICC) and coefficient of variance (CV) were used to analyze intra-session reliability. The highest flight time, jump height and power were used for inter-session analysis. Pearson’s correlation (r_p_), the ICC and CV were used to analyze the inter-session reliability. As part of the inter-session reliability analysis, the standard error of the measurement (SEM) was assessed and calculated as SEM = SD × √(1-ICC) [14]. To analyze the minimum meaningful change between measurements, the minimum detectable change (MDC) was used and calculated according as MDC = SEM × 1.96 × √2 [14]. Also the relative MDC (MDC%) was calculated as MD divided by the mean of all observations. Measurement error (ME) [14] was calculated as the standard deviation of the difference scores between test and retest divided by the root square of two and typical error (TE%) [15] was calculated as ME divided by the mean of all test results.

The following methods for assessing the coherence between M-encoder, IR-mat, and C-mat were used: 1) Mean difference and standard deviation with a 2 (methods) x 3 (trails) repeated measures ANOVA approach to detect statistical significant differences; 2) Pearson’s correlations were used to analyze strength of associations between methods; 3) M-encoder, IR-mat, and C-mat were compared two and two in a Bland-Altman analysis to find any systematic variance [16]. The p < 0.05 criterion was used for establishing statistical significance; and 4) Limits of Agreement (LOA) was calculated for the Bland-Altman plots to show upper and lower LOA [16].

### Ethics

Ethical principles outlined in the declaration of Helsinki were followed. This study was approved by the Ethics Committee in Lund, Sweden (ETIK 2009/699).

## Results

All data, except for power assessed with the M-encoder on the second session (p = 0.014), were normally distributed according to the Kolmogorov-Smirnov test. Since no main effect was seen by session between the men and women (Table 1), the groups were analyzed as one group to increase the statistical power of the calculations.

**Table 1:** **Two (genders) x two (sessions) repeated measurement ANOVA analysis of two-legged CMJ with external loading of 40 kg**

**Methods**	**Effect**	**F**	**df**	**P**	**Post hoc**	**P**
*C-mat (Flight time)*	Ge x Ses					
	Ge	46.6	1.0	<0.001	M > W	<0.001
	Ses	1.7	1.0	ns		
*C-mat (Jump height)*	Ge x Ses					
	Ge	54.8	1.0	<0.001	M > W	<0.001
	Ses	1.2	1.0	ns		
*C-mat (Power)*	Ge x Ses					
	Ge	60.3	1.0	<0.001	M > W	<0.001
	Ses	3.1	1.0	ns		
*IR-mat (Flight time)*	Ge x Ses					
	Ge	57.9	1.0	<0.001	M > W	<0.001
	Ses	0.0	1.0	ns		
*IR-mat (Jump height)*	Ge x Ses					
	Ge	60.8	1.0	<0.001	M > W	<0.001
	Ses	0.3	1.0	ns		
*M-encoder (Power)*	Ge x Ses					
	Ge	58.4	1.0	<0.001	M > W	<0.001
	Ses	0.5	1.0	ns		

CMJ = Counter movement jump; Ge = Gender; Ses = Session; M = men; W = women.

The variables analyzed were Flight time (ms) and jump height (cm) for C-mat and IR-mat, and power (W) for C-mat and M-encoder.

No significant differences between the three jump trails during session 1 were found (Table 2). Through the post hoc analysis was a significant differences between C-mat and IR-mat assessing jump height (p < 0.001), and C-mat vs M-encoder assessing power (p < 0.001) demonstrated.

**Table 2:** **Two (methods) x three (trials) repeated measurement ANOVA analysis of two-legged CMJ with external loading of 40 kg**

**Methods**	**Effect**	**F**	**df**	**P**	**Post hoc**	**P**
*C-mat vs IR-mat (Jump height)*	Tr	1.1	2.0	ns		
	Met	313.0	1.0	<0.001	C-mat > IR-mat	<0.001
*C-mat vs M-encoder (Power)*	Tr	3.1	1.9	ns		
	Met	1411.0	1.0	<0.001	C-mat > M-encoder	<0.001

CMJ = Counter movement jump; Tr = Trial; Met = method.

The variables analyzed were jump height (cm) and power (W).

The intra-session reliability was high within each assessment method (Table 3), with an ICC ranging from 0.97 to 1.00 and with a CV from 1.0% to 5.3%. The inter-session reliability was also high (Table 2), with an ICC ranging from 0.94 to 0.99 and with a CV from 1.7% to 6.1%. The SEM was almost identical for flight time and jump height assessed with C-mat and IR-mat and somewhat higher assessing power with M-encoder compared to C-mat. The MDC was 39 ms for flight time assessed with both C-mat and IR-mat, while MDC was 3.9 cm and 1.5 cm for jump height assessed with C-mat and IR-mat respectively. During assessments of power the MDC was 80 W for C-mat and 87 W for M-encoder.

**Table 3:** **Inter-session reliability during session 1 and session 2**

	**Session 1**	**Session 2**	**Intra-session reliability**			**Inter-session reliability**				
	**Mean (SD)**	**Mean (SD)**	**CV (95% CI)**	**ICC (95% CI)**	**r**_**p**_	**CV (95% CI)**	**ICC (95% CI)**	**SEM**	**MDC**	**MDC%**
*C-mat*										
Flight Time (ms)	404 (60)	396 (58)			0.94	2.6 (2.0-3.3)	0.94 (0.90-0.97)	14	39	9.8
Jump Height (cm)	19.9 (6.0)	19.7 (5.7)	3.6 (2.6-4.6)	0.97 (0.95-0.98)	0.94	5.7 (4.2-7.3)	0.94 (0.88-0.97)	1.4	3.9	19.6
Power (W)	4387 (807)	4325 (788)	1.0 (0.7-1.2)	1.00 (0.99-1.00)	0.99	1.7 (1.3-2.2)	0.99 (0.97-0.99)	80	222	5.1
*IR-mat*										
Flight Time (ms)	364 (69)	369 (66)			0.96	3.0 (2.0-40)	0.96 (0.92-0.98)	14	39	10.6
Jump Height (cm)	16.9 (6.9)	17.1 (6.1)	5.3 (4.0-6.6)	0.98 (0.97-0.99)	0.96	6.1 (4.1-8.2)	0.96 (0.93-0.98)	1.3	1.5	8.8
*M-encoder*										
Power (W)	1650 (392)	1663 (381)	2.8 (2.2-3.3)	0.98 (0.97-0.99)	0.97	2.9 (2.1-3.8)	0.97 (0.95-0.98)	87	241	9.1

CMJ = Counter movement jump; CV = Coefficient of variation; CI = Confidence interval; ICC = Intraclass correlation coefficient; SEM = Standard error of the measurement; MDC = Minimal detectable change; r_p_ = Pearsson’s correlation coefficient.

Two-legged and one-legged CMJ with external loading in kg. The variables were analyzed as power (W).

Test-retest data from measurement with M-encoder, in Table 4, show the correlation and comparison between session 1 and session 2 for AP, and the statistical parameters and reliability coefficients (r_p_, ME, and TE%) for all variables and loads. The assessments of AP show that there was a significant and strong correlation (r_p_ = 0.97, p < 0.001) between session 1 and 2. TE%, which is a value of the relative spread or ME, was 3.9%. The correlation and comparison between session1 and session 2 for AF assessed with M-encoder (Table 4) showed a significant and strong correlation (r_p_ = 0.99, p < 0.001). The value for TE% (1.4%) was low. There was a strong and significant correlation (r_p_ = 0.94, p < 0.001) for assessments of AV between session1 and session 2 (Table 4). TE% for AV was 2.9%. The correlation between session 1 and session 2 for D (Table 4), also showed a high and significant correlation (r_p_ = 0.87, p < 0.001). TE% for D showed the highest values, of 5.4%. Knee angle had the lowest relation between session 1 and session 2 (r_p_ = 0.51, p < 0.001) with a TE of 4.5% (Table 4).

**Table 4:** **Results of counter movement jump session 1 and session 2**

	**Session 1**	**Session 2**	**r**_**p**_	**ME**	**TE%**
*M-encoder*					
AP (W)	1650 (392)	1663 (381)	0.97	64.8	3.9
AF (N)	1396 (172)	1400 (173)	0.99	19.0	1.4
AV (m/s)	1.17 (0.14)	1.18 (0.14)	0.94	0.03	2.9
D (cm)	57 (9)	57 (9)	0.87	3.1	5.4
Knee angle (degrees)	101 (6)	102 (7)	0.51	4.5	4.5

AP = Average power; AF = Average force; AV = Average velocity; D = Distance; ME = Measure error; TE% = Typical error.

M-encoder on AP, AF, AV, D and knee angle. Indices of test-retest reliability, Pearson’s correlation coefficient, ME and TE% are reported for M-encoder on AP, AF, AV, D and knee angle, on load 40 kg.

Mean jump height and flight time assessed with the C-mat and the IR-mat, and power assessed with the C-mat and the M-encoder are reported in Table 3. Significant differences were demonstrated between the C-mat and the IR-mat assessing jump height and flight time and between the C-mat and the M-encoder assessing power in a 2x3 ANOVA approach (Table 2).

**Figure 1:**
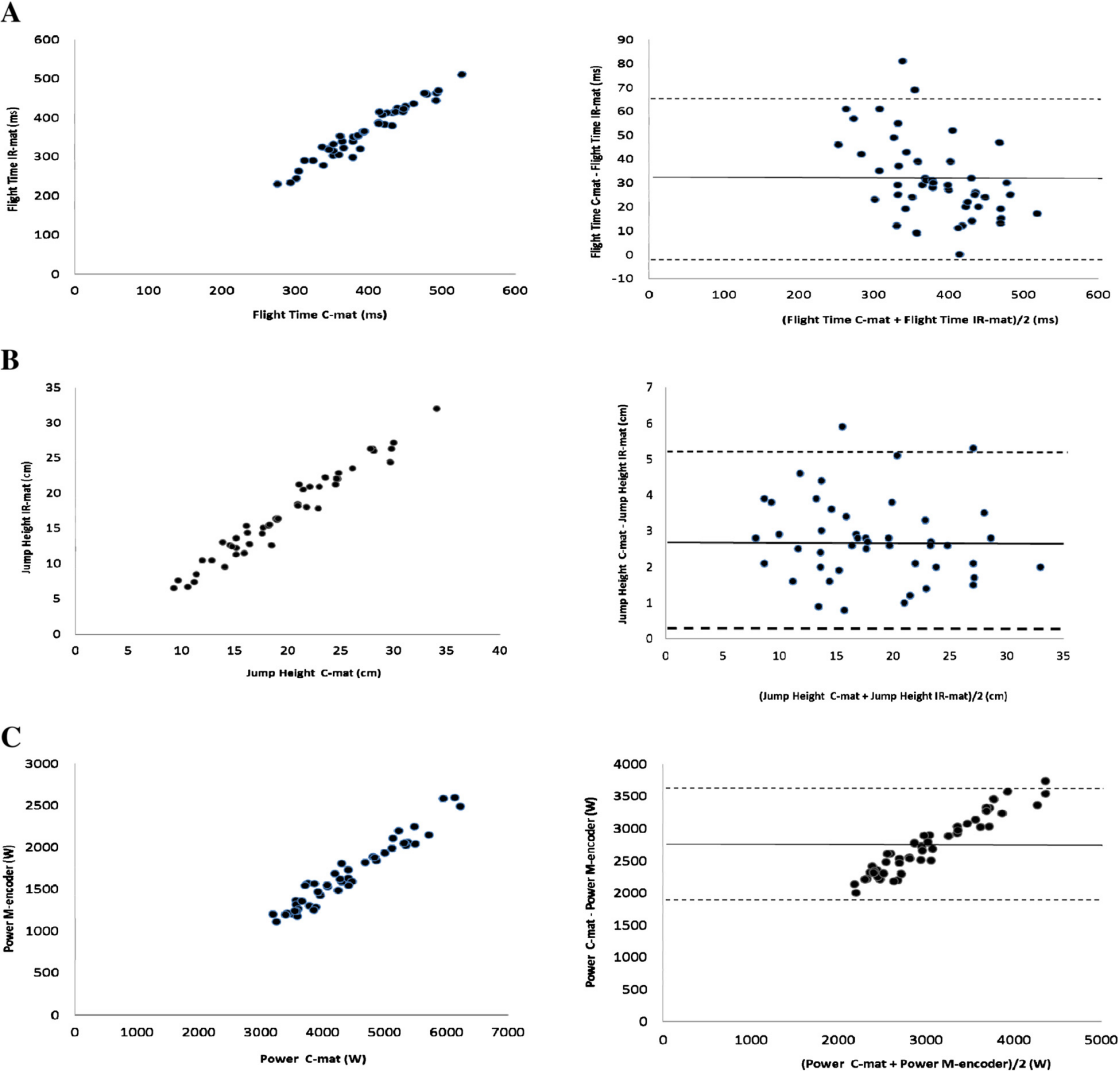
Shows correlation plots and Bland Altman plots for relation analysis between C-mat, IR-mat and M-encoder. **A.** Shows correlation plots for relation analysis between C-mat and IR-mat for flight time (ms) with the external load of 40 kg (r_p_ = 0.97, p < 0.001) and Bland Altman plots, comparison between C-mat and IR-mat for flight time (ms) with the external load of 40 kg (mean difference 31.6 ms, LOA = 33.6 ms). **B.** Shows correlation plots for relation analysis between the C-mat and IR-mat for jump height (cm) with the external load of 40 kg (r_p_ = 0.98, p < 0.001) and Bland Altman plots, comparison between C-mat and IR-mat for jump height (cm) with the external load of 40 kg (mean difference 2.7 cm, LOA = 2.4 cm). **C.** Shows correlation plots for association between C-mat and M-encoder for power (watt) with the external load of 40 kg (r_p_ = 0.97, p < 0.001) and Bland Altman plots, comparison between C-mat and M-encoder for power (W) with the external load of 40 kg (mean difference = 2726 W, LOA = 888 W).

In the coherence analysis for flight time assessed with C-mat and IR-mat a significant relationship (r_p_ = 0.97, p < 0.001) was found (Figure 1). In the Bland Altman plots analysis, was a systematic bias between the C-mat and IR-mat assessments found, with a mean difference of 31.6 ms and LOA of 33.6 ms. Also a significant relation (r_p_ = 0.98, p < 0.001) was between the C-mat and IR-mat for jump height and the Bland Altman plots demonstrated a mean difference of 2.7 cm with a LOA of 2.4 cm between assessments of jump height with C-mat and IR-mat. When comparing assessments of power with C-mat and M-encoder a significant relation was found (r_p_ = 0.97, p < 0.001) and a systematic bias with a mean difference of 2726 W and a LOA of 888 W.

## Discussion

The main finding of this study was that there is a high reproducibility of M-encoder, IR-mat and C-mat assessing CMJ performance among elite athletes. We have also shown that the power assessments obtained from the C-mat were systematically higher than the ones from the M-encoder.

Elite athletes need to test their progress of training on a regular basis and it is important that the test methodology is reliable and has a small within-subject variation [15] in order to detect changes. A high correlation coefficient, according to Atkinson and Nevill [14], is values above 0,8. Hori et al. [9] and Carlock et al. [6] define values of Pearson’s correlation coefficient r_p_ > 0.9 as nearly perfect, 0.7-0.9 as very high, 0.5-0.7 as high, 0.3-0.5 as moderate, 0.1-0.3 as small, and 0.1 or less as trivial. In light of this, the correlation data in our study are nearly perfect or very high apart from the values for knee angle that was moderate (Tables 3 and 4).

This study was designed to investigate the coherence of three different testing systems. The main equipment, the C-mat, was compared with both the M-encoder and IR-mat. There was strong coherence between both the C-mat and M-encoder and the C-mat and IR-mat. But, the difference obtained between the C-mat and M-encoder depends presumably on two different algorithms to calculate the power outcome. Also the construct of the contact mat and the encoder could presumably contribute to the differences between the two assessment methods. In contrast, the difference between the C-mat and IR-mat was more peculiar. The contact mat raised 6 mm above the ground and the IR-mat 16 mm above the ground, which could give a systematic bias since the starting point of assessment differs between the C-mat and the IR-mat. However, this does not explain the whole variation. Both the C-mat and the IR-mat used the same algorithm to calculate jump height, which is based on flight time. The flight time was measured by the C-mat from the moment the subject takes off from the mat to the moment the subject lands. The IR-mat flight time was measured from the moment the subject takes off and the infrared beam was switched off, until the subject lands again, at which time the infrared beam was switched on. The mean difference between the C-mat and the IR-mat was approximately 3 cm for jump height in our study, similar to the results obtained by Enoksen et al. [10] for jump height, 2.8 cm. Since these differences are larger that the SEM for both C-mat and IR-mat, it is not acceptable for clinical purposes to use the C-mat and IR-mat interchangeably [10]. In coherence with Enoksen et al. [10], we also demonstrate the importance of always using the same assessment equipment during pre- and post-testing.

The overall results show that the test-retest reliability was good, since the values for Pearson’s correlations coefficient and ICC between session 1 and session 2 were high. MDC%, TE% and LOA were low for the indices analyzed, except for MDC% of jump height assessed with C-mat (Table 3). Meaning that the athlete needs to improve its performance by approximately 19% before it can be considered at true change. From this point of view the C-mat has a poor discriminative capacity. As the reliability of a test influences the accuracy of a single measure it is important that all equipment used for testing athletes are reliable. Otherwise, the athletes would not be able to track their changes in performance over time [2]. Earlier studies have reported TE% values below 10% as reliable [11,17] and the fact that the TE% values (<6%) were low implies that CMJ with external load assessed with the M-encoder is a test capable of evaluating the progression of training of power with high reproducibility, even when it comes to minor changes in performance.

Atkinson and Nevill [14] discuss systematic bias that is associated with ME, which affects the TE% values. Systematic bias refers to a general trend for measurements between repeated tests. The trend can either show that the retest values are better due to a learning effect or that the retest values are worse due to insufficient recovery between tests. Since no significant differences were found in the post hoc analysis of the ANOVA analysis between session 1 and session 2, no systematic bias between the sessions seemed to have been apparent. This could be explained by the fact that there were three days between trials, which would be enough according to Atkinson and Nevill [14] who claim that exercise performance tests need more than one day in between repeated measurements for adequate recovery. A learning effect from session 1 to session 2 may have been avoided in the present study, since the participants performed three test jumps before the actual test started and that these elite athletes were use to vertical jumping. Even though the subjects in our study were elite athletes use to perform vertical jumps, familiarizations trails are important as Hopkins [15] discusses the learning effect in his study and suggests that in order to avoid learning effects, familiarization trials should be allowed.

## Conclusion

All the three assessment methods were reliable but not interchangeable. Assessments of flight time and jump height gave higher values assessed with the C-mat compared to the IR-mat in ms and cm respectively. Also the power assessments with the C-mat gave higher values compared to the M-encoder in watts.

The results from the present study show that CMJ with external load assessed M-encoder is reliable. This knowledge will be of great interest to athletes and practitioners who use these tools. Athletes and practitioners will be able to carry out reliable tests and evaluate physical improvements, knowing that results are due to training and not due to variance in the test methodology.

## Abbreviations

CMJ: Counter movement jump
P: Power
F: Force
V: Velocity
AP: Average power
AF: Average force
AV: Average velocity
RM: Repetition maximum
SD: Standard deviation
ME: Measure error
CV: Coefficient of variance
r_p_: Pearson’s correlation coefficient
LOA: Limits of agreement
ICC: Intraclass correlation coefficient
MDC: Minimum detectable change
MDC%: Relative MDC
SEM: Standard error of the measurement
TE%: Typical error
